# Modulatory Effect of Eui-E-In-Tang on Serum Leptin Concentration in Obese Korean Female Adults: A Randomized Controlled Trial

**DOI:** 10.1155/2016/1894837

**Published:** 2016-08-18

**Authors:** Yun-Kyung Song, Ji-Young Lee, Wansu Park

**Affiliations:** ^1^Department of Korean Rehabilitation Medicine, College of Korean Medicine, Gachon University, Seongnam 13120, Republic of Korea; ^2^Department of Pathology, College of Korean Medicine, Gachon University, Seongnam 13120, Republic of Korea

## Abstract

*Background.* Obesity is associated with chronic inflammation and cytokines. However, to date, the relationship between the serum levels of cytokines in obese individuals and taking herbal drugs remains largely unexplored.* Methods.* Serum cytokines were assessed by multiplex cytokine profiling assay. Serum samples of obese female Korean adults (obese group; *n* = 20) as well as normal female Korean adults (normal group; *n* = 21) were collected at the start and end of study period. Twenty obese female Korean adults were randomized to receive Eui-E-In-Tang (Eui-E-In-Tang group; *n* = 9) at a daily dose of 9 g or a matched placebo (placebo group; *n* = 11) for 12 weeks.* Results.* According to investigating serum cytokine levels at the start point of this study, the serum C-peptide, insulin, leptin, lipocalin-2, and adipsin levels in the obese group were found to be significantly higher than in the normal group. And the investigation of serum cytokine levels at the end point of this study demonstrated that mean serum leptin of Eui-E-In-Tang group was found to be significantly reduced (*P* = 0.037).* Conclusions.* This study provides preliminary evidence that Eui-E-In-Tang may exert immunomodulatory effect via altering the circulating concentration of leptin in Korean female adults.

## 1. Introduction

Obesity is a serious health problem worldwide. According to the World Health Organization (WHO) statistics, there were more than 1.9 billion overweight adults globally, of whom about 600 million were obese in 2014 [1].

Obesity is associated with an increased risk of life-threatening diseases including diabetes, cardiovascular diseases, metabolic syndrome, osteoarthritis, gout, and certain types of cancer [2–4]. Adya et al. have already reported that obesity is a major health burden with an increased risk of cardiovascular morbidity and mortality [5]. dos Santos et al. have reported that inflammation, the process aimed at restoring homeostasis after an insult, can be more damaging than the insult itself if uncontrolled, excessive, or prolonged [6]. Specifically, in mediators of chronic inflammation, cytokine (cell signaling protein) has been becoming the impressive target to deal with obesity. Alvehus et al. have reported that obesity can be considered as a low-grade inflammatory condition, strongly linked to adverse metabolic outcomes [7]. It has been suggested that adipocytokine (adipokine) such as leptin is secreted by adipose tissue and concerned with the pathogenesis of obesity-associated complications [8, 9].

Yen et al. have reported that inflammation has been found to be an important characteristic of adipose tissue in obese subjects and obesity is also associated with compromised immune responses to infections, which have not been fully understood [10]. Schulte et al. have reported that cytokines are important pleiotropic regulators of the immune response, which have a crucial role in the complex pathophysiology underlying sepsis [11]. Recently, anti-inflammatory effects of herbal drugs used in Korea, China, and Japan have been reported continuously. Eui-E-In-Tang is one of herbal drugs which are used for obese individuals in Korea.

However, to date, the relationship between the serum levels of cytokines in obese individuals and taking herbal drug such as Eui-E-In-Tang remains largely unexplored, especially in Korean women. In this study, we investigated modulatory effects of Eui-E-In-Tang on serum concentrations of cytokines in obese female Korean adults.

## 2. Materials and Methods

### 2.1. Study Design

This was a randomized, double blind, placebo-controlled trial in which each patient received Eui-E-In-Tang (9 g/day) or a matched placebo for 12 weeks. All studies were approved by Bioethic Institutional Review Board at Gachon University. All subjects gave written informed consent prior to participation.

### 2.2. Subjects

Thirty-six apparently healthy adult Korean females were recruited from the local communities of Incheon and neighboring areas as normal subjects (the normal group). Obesity was defined by body mass index (BMI) ≥ 30.0 kg/m^2^. Thirty-six obese adult Korean females were selected and recruited from the local communities of Incheon and neighboring areas as obese subjects (the obese group). Thirty-one individuals were excluded if they were taking any prescription or over-the-counter medication including oral contraceptives, hormone replacement therapy, or psychiatric drugs or had any acute or chronic medical illnesses during the study. Finally, 21 normal subjects and 20 obese subjects were selected for serum cytokine profiling assay in the study.

### 2.3. Drugs

Eui-E-In-Tang and placebo drug capsules were provided by the Hanpoong Pharmaceutical Co., Ltd. (Seoul, Korea). The components of Eui-E-In-Tang capsule are shown in Table 1. The placebo capsules were made of cornstarch and manufactured identical to Eui-E-In-Tang capsules in terms of color, size, and shape by the same manufacturer.

### 2.4. Multiplex Cytokine Assay

Multiplex cytokine profiling assay (MILLIPLEX MAP Human Metabolic Panel, Millipore, Billerica, MA, USA) was used to simultaneously measure serum levels of 21 cytokines such as C-peptide, ghrelin, glucose-dependent insulinotropic polypeptide (GIP), glucagon-like peptide-1 (GLP-1), glucagon, interleukin- (IL-) 1*β*, IL-6, IL-8, insulin, leptin, monocyte chemotactic protein- (MCP-) 1, pancreatic polypeptide (PP), peptide YY (PYY), tumor necrosis factor- (TNF-) *α*, nerve growth factor (NGF), hepatocyte growth factor (HGF), adiponectin, lipocalin-2, resistin, adipsin, and total plasminogen activator inhibitor type-1 (PAI-1). Samples were read on a Bio-Plex 200 suspension array system (Bio-Rad, Hercules, CA, USA) using Bio-Plex Manager 5.0 software system (Bio-Rad). The standard curve range for each cytokine was from 3.2 to 10,000 pg/mL.

### 2.5. Statistical Analysis

The results represent the mean ± SD. Descriptive analyses for the normal and obese groups are done using the independent sample *t*-test. The serum cytokine levels of the normal and obese groups were also compared via an independent sample *t* test. Of them, C-peptide, insulin, leptin, HGF, adiponectin, lipocalin-2, adipsin, and total PAI-1—all of which showed significant differences between the two groups—were compared with a univariate analysis of covariance. Correlation coefficients were calculated using Spearman's test. A paired *t*-test was used to compare serum cytokine levels before and after Eui-E-In-Tang therapy. All tests were two-tailed at a probability level of 0.05. All analyses were performed using SPSS software 19.0 (SPSS Inc., Chicago, IL, USA).

## 3. Results

Table 2 presents the demographic factors, height, weight, and body mass index of the individuals in the normal and obese groups. We also compared vascular risk factors between the normal and obese group. Significant differences in the serum glutamate-pyruvate transaminase (SGPT), alkaline phosphatase, gamma-glutamyl transpeptidase (GGTP), total cholesterol, high-density lipoprotein (HDL) cholesterol, triglyceride, uric acid, glucose, C-reactive protein (CRP), and white blood cell (WBC) levels were observed between the two groups.

Among the 21 cytokines, the number of samples with detectable levels and the range (pg/mL) varied according to the tested cytokines. Data of cytokines which were detected to be >80% of the total samples were used for further analysis and presented in Table 3. Data of cytokines included C-peptide, GIP, insulin, leptin, MCP-1, PP, IL-8, HGF, adiponectin, lipocalin-2, resistin, adipsin, and total PAI-1. Among the 13 cytokines analyzed, the serum C-peptide, insulin, leptin, HGF, lipocalin, adipsin, and total PAI-1 levels of the obese group were found to be significantly higher than those of the normal group (*t* = −5.104, *P* < 0.001; *t* = −3.906, *P* = 0.001; *t* = −6.777, *P* < 0.001; *t* = −2.753, *P* = 0.01; *t* = −2.062, *P* = 0.046; *t* = −3.723, *P* = 0.001; and *t* = −3.441, *P* = 0.001, resp.), whereas the serum adiponectin level of the obese group was found to be lower than that of the normal group (*t* = 2.77, *P* = 0.009) (Table 3). After adjusting for age, height, SGPT, alkaline phosphatase, GGTP, total cholesterol, HDL cholesterol, triglyceride, uric acid, glucose, CRP, and WBC, the serum C-peptide, insulin, leptin, lipocalin-2, and adipsin levels in the obese group were found to be significantly higher than in the normal group (*F* = 4.673, *P* = 0.04; *F* = 4.358, *P* = 0.046; *F* = 28.469, *P* < 0.001; *F* = 7.236, *P* = 0.012; *F* = 5.37, *P* = 0.028, resp.).

Table 4 presents correlations among serum cytokines in the overall population. C-peptide was significantly correlated with insulin, leptin, IL-8, HGF, adiponectin, adipsin, and total PAI-1. GIP was significantly correlated with insulin, adiponectin, and lipocalin-2. Insulin was significantly correlated with leptin, HGF, adipsin, and total PAI-1. Leptin was significantly correlated with HGF, lipocalin-2, adipsin, and total PAI-1. PP was significantly correlated with adiponectin, resistin, and total PAI-1. IL-8 was significantly correlated with lipocalin-2. HGF was significantly correlated with lipocalin-2, resistin, adipsin, and total PAI-1. Lipocalin-2 was significantly correlated with resistin and adipsin. Resistin was significantly correlated with adipsin. However, MCP-1 was not significantly correlated with any other cytokines.

Table 5 presents that mean serum leptin of Eui-E-In-Tang group was found to be significantly reduced by Eui-E-In-Tang therapy for 12 weeks (*P* = 0.037). In contrast, Eui-E-In-Tang did not alter serum levels of C-peptide, GIP, insulin, MCP-1, PP, IL-8, HGF, adiponectin, lipocalin-2, resistin, adipsin, and total PAI-1 (*P* > 0.05). Table 5 also presents that the serum cytokine levels of obese group with the placebo capsules for 12 weeks do not show any significant variation (*P* > 0.05).

## 4. Discussion

Overweight and obesity are defined as abnormal or excessive fat accumulation that may impair health by the WHO definition [1]. Obesity is a global health problem and is associated with chronic inflammation, which is linked to many diseases including diabetes, cardiovascular diseases, metabolic syndrome, and gout. According to the WHO statistics, about 13% of the world's adult population (11% of men and 15% of women) was obese in 2014 [1].

Ghayour-Mobarhan et al. have reported that obesity is associated with a strong inflammatory response and is often accompanied by increased levels of proinflammatory cytokines and impaired antioxidant status [12]. Spite et al. have reported that although inflammation is thought to be essential for reacting to pathologic infection, ungoverned inflammation is an underlying component of various diseases, such as sepsis, cardiovascular disease, diabetes, and other chronic inflammatory diseases [13]. Maskrey et al. have reported that chronic inflammation is a characteristic feature in virtually all inflammatory diseases, including cardiovascular disease such as atherosclerosis, and it is becoming increasingly clear that disturbance of the processes usually involved in resolution of inflammation is an underlying feature of chronic inflammatory conditions [14]. Skeldon et al. have reported that inflammation also is regarded as an important contributor to the development of metabolic disease and recent work has strongly implicated the inflammasome as having a pivotal role in the regulation of metabolism, obesity, insulin resistance, and cardiovascular disease [15].

It is well known that cytokine is an important mediator in acute or chronic inflammation. Burska et al. have reported that cytokines are small proteins which play important roles in cell signaling and they are secreted by a variety of cellular sources acting either on the cell producing them (autocrine) or on the surrounding cells (paracrine) [16]. Nakagawa et al. have reported that IL-6 is a multifunctional cytokine that is produced by many different cell types, and plays an important role in the regulation of inflammation, immune responses, the acute-phase response, and hematopoiesis [17]. Niebler et al. have reported that IL-1*β* is also included in central key players in the immune surveillance interactome, which not only mediates inflammation but also links innate and adaptive immunity [18]. Tracey et al. have reported that TNF-*α* is a pleiotropic cytokine known to play a major role in host defense mechanisms, initiating a beneficial local inflammation which in excess, however, may cause tissue damage concerned with the pathogenesis of numerous autoimmune and/or inflammatory systemic diseases [19].

Cao has reported that obesity induces production of inflammatory cytokines (often referred to together with adipokines as adipocytokines) and infiltration of immune cells into adipose tissue, which creates a state of chronic low-grade inflammation [20]. Ganjali et al. have reported that the most important source of proinflammatory cytokines in obesity is macrophages that infiltrate adipose tissue as a response to the adipocyte growth, decreased blood supply, hypoxia, and tissue necrosis [21]. Blüher and Mantzoros have reported that the adipose tissue influences the regulation of several important physiological functions including but not limited to appetite, satiety, energy expenditure, activity, insulin sensitivity and secretion, glucose and lipid metabolism, fat distribution, endothelial function, hemostasis, blood pressure, neuroendocrine regulation, and function of the immune system through adipokines [9]. On the other hand, Park et al. have reported that Euiiin-tang (yiyiren-tang; Eui-E-In-Tang) granules exert anti-inflammation and antiobesity effect on high fat diet-induced obese C57 BL/6J mice [22].

These days, the number of reports for adipokines with obesity is increasing. Leptin is well known to be a forerunner of the adipokine superfamily. Park and Ahima have reported that leptin is secreted by adipose tissue and regulates energy homeostasis, neuroendocrine function, metabolism, immune function, and other systems through its effects on the central nervous system and peripheral tissues [8].

In order to examine the inflammatory mediators of obesity and determine a possible distinctive inflammatory blood marker, we measured the serum levels of 21 cytokines using multiplex cytokine assay. Unlike infectious inflammation diseases increase the serum levels of proinflammatory cytokines such as IL-1*β*, IL-6, TNF-*α*, and MCP-1, our data represent that the serum C-peptide, insulin, leptin, HGF, lipocalin, adipsin, and total PAI-1 levels are higher in the obese group than the normal group; the serum adiponectin level is lower in the obese group than the normal group. After adjusting for age, height, SGPT, alkaline phosphatase, GGTP, total cholesterol, HDL cholesterol, triglyceride, uric acid, glucose, CRP, and WBC, the serum C-peptide, insulin, leptin, lipocalin-2, and adipsin levels in the obese group were found to be significantly higher than in the normal group. C-peptide was significantly correlated with insulin, leptin, IL-8, HGF, adiponectin, adipsin, and total PAI-1. Insulin was significantly correlated with C-peptide, GIP, leptin, HGF, adipsin, and total PAI-1. Leptin was significantly correlated with C-peptide, insulin, HGF, lipocalin-2, adipsin, and total PAI-1. Lipocalin-2 was significantly correlated with GIP, leptin, IL-8, HGF, resistin, and adipsin. Adipsin was significantly correlated with C-peptide, insulin, leptin, HGF, lipocalin-2, and resistin. How correlations of cytokines are varied and involved in obesity deserves further investigation in cells, animal models, and human populations.

And the investigation of serum cytokine levels at the end point of this study demonstrated that mean serum leptin of Eui-E-In-Tang group was found to be significantly reduced by Eui-E-In-Tang therapy for 12 weeks. In contrast, Eui-E-In-Tang therapy did not show any significant effect on the concentrations of C-peptide, insulin, lipocaline-2, adipsin, HGF, total PAI-1, GIP, MCP-1, PP, IL-8, adiponectin, and resistin. The findings on the modulatory effect of Eui-E-In-Tang on serum level of leptin deserve to be studied further to make a safe and effective treatment for obesity.

We acknowledge limitations in our study. The most important limitation relates to the small sample size. Another limitation of the present study was the lack of gender diversity in the study group. It is recommended that further studies be designed for a large sample size including male individuals.

## Figures and Tables

**Table 1:** Composition of Eui-E-In-Tang.

Herbal medicines	Ratio
Coicis Semen	10
Angelicae Gigantis Radix	4
Atractylodis Rhizoma Alba	4
Ephedrae Herba	4
Cinnamomi Ramulus	3
Paeoniae Radix	3
Glycyrrhizae Radix	2

**Table 2:** Demographic characteristics and obesity-related scale scores of participants.

	Normal group (*n* = 21, female)	Obese group (*n* = 20, female)	*t*	*P*
Age (year)	36.7 ± 11.1	41.4 ± 9.5	−1.436	0.159
Height (cm)	161.5 ± 4.7	160.2 ± 6.2	0.796	0.431
Weight (kg)	53.8 ± 4.4	87.6 ± 12.2	−11.729	<0.001
Body mass index (BMI)	20.7 ± 1.5	34.0 ± 3.1	−17.397	<0.001
Total protein (g/dL)	7.5 ± 0.3	7.3 ± 0.5	1.018	0.315
Albumin (g/dL)	4.7 ± 0.2	4.6 ± 0.2	1.419	0.164
Total bilirubin (g/dL)	0.7 ± 0.3	0.6 ± 0.3	1.257	0.216
SGOT (U/L)	19.2 ± 6.8	24.4 ± 10.1	−1.939	0.06
SGPT (U/L)	15.4 ± 8.9	25.0 ± 11.5	−2.971	0.005
Alkaline phosphatase (U/L)	49.6 ± 14.3	67.4 ± 16.4	−3.691	0.001
GGTP (U/L)	14.4 ± 6.8	33.0 ± 28.6	−2.839	0.01
Creatine phosphokinase (U/L)	111.4 ± 123.8	98.8 ± 66.6	0.404	0.688
Total cholesterol (mg/dL)	174.1 ± 24.3	201.7 ± 46.7	−2.359	0.025
HDL cholesterol (mg/dL)	62.1 ± 14.5	53.2 ± 13.0	2.061	0.046
Triglyceride (mg/dL)	74.8 ± 32.8	133.5 ± 60.7	−3.822	0.001
Uric acid (mg/dL)	4.4 ± 0.8	5.6 ± 1.3	−3.675	0.001
Blood urea nitrogen (mg/dL)	11.8 ± 2.4	12.8 ± 3.7	−1.045	0.303
Creatinine (mg/dL)	0.7 ± 0.1	0.7 ± 0.1	−0.737	0.465
Glucose (mg/dL)	85.8 ± 9.1	93.4 ± 10.2	−2.512	0.016
CRP (mg/dL)	0.0 ± 0.1	0.3 ± 0.5	−2.533	0.02
Free T4 (ng/dL)	1.2 ± 0.1	1.2 ± 0.2	−0.573	0.57
Thyroid-stimulating hormone (*μ*IU/mL)	1.6 ± 0.7	1.6 ± 0.9	−0.141	0.888
Hemoglobin (g/dL)	13.3 ± 1.0	13.6 ± 0.9	−1.111	0.273
Hematocrit (%)	40.3 ± 2.6	41.1 ± 2.1	−1.069	0.291
RBC count (10^6^/*μ*L)	4.4 ± 0.4	4.5 ± 0.3	−1.384	0.174
WBC count (10^3^/*μ*L)	5.7 ± 1.8	7.1 ± 1.8	−2.408	0.021
Platelet count (10^3^/*μ*L)	268.1 ± 58.8	279.2 ± 46.7	−0.668	0.508

**Table 3:** Comparison of serum cytokine levels using multiplex cytokine assays.

Serum cytokine levels (pg/mL)	Normal (*n* = 21, female)	Obese (*n* = 20, female)	*t*	*P*
C-peptide	1673.2 ± 787.3	3395.5 ± 1319.7	−5.104	<0.001
GIP	64.4 ± 54.3	102.5 ± 203.1	−0.831	0.411
Insulin	27.7 ± 13.7	52.4 ± 24.9	−3.906	0.001
Leptin	842.0 ± 706.2	3498.7 ± 1612.0	−6.777	<0.001
MCP-1	1163.7 ± 493.1	1313.7 ± 430.1	−1.036	0.307
PP	110.8 ± 61.8	119.2 ± 72.1	−0.402	0.69
IL-8	2098.1 ± 2500.2	1637.5 ± 3468.3	0.49	0.627
HGF	174.1 ± 84.0	285.3 ± 160.8	−2.753	0.01
Adiponectin	10228.4 ± 1709.2	8732.5 ± 1748.4	2.77	0.009
Lipocalin-2	3429.9 ± 1335.8	4552.5 ± 2086.3	−2.062	0.046
Resistin	807.3 ± 530.7	1048.1 ± 669.4	−1.28	0.208
Adipsin	16287.9 ± 1044.8	17351.4 ± 752.5	−3.723	0.001
Total PAI-1	1953.5 ± 647.3	2701.9 ± 743.7	−3.441	0.001

**Table 4:** Spearman correlation analysis of cytokines.

	C-peptide	GIP	Insulin	Leptin	MCP-1	PP	IL-8	HGF	Adiponectin	Lipocalin-2	Resistin	Adipsin	Total PAI-1
C-peptide	1	0.306	0.852^*∗∗*^	0.651^*∗∗*^	−0.029	−0.017	−0.328^*∗*^	0.418^*∗∗*^	−0.407^*∗∗*^	0.274	0.176	0.318^*∗*^	0.634^*∗∗*^
GIP		1	0.409^*∗∗*^	0.261	−0.232	0.033	−0.153	0.084	0.312^*∗*^	0.364^*∗*^	−0.017	0.179	0.273
Insulin			1	0.583^*∗∗*^	−0.036	−0.089	−0.275	0.471^*∗∗*^	−0.201	0.287	0.111	0.374^*∗*^	0.587^*∗∗*^
Leptin				1	0.158	0.075	−0.082	0.536^*∗∗*^	−0.282	0.322^*∗*^	0.12	0.414^*∗∗*^	0.615^*∗∗*^
MCP-1					1	0.115	0.052	0.083	−0.007	−0.15	−0.12	−0.078	0.06
PP						1	−0.027	−0.063	0.35^*∗*^	−0.04	0.409^*∗∗*^	0.093	0.383^*∗*^
IL-8							1	0.276	0.155	0.422^*∗∗*^	0.289	−0.085	−0.272
HGF								1	−0.302	0.635^*∗∗*^	0.358^*∗*^	0.484^*∗∗*^	0.422^*∗∗*^
Adiponectin									1	−0.032	−0.033	−0.119	−0.041
Lipocalin-2										1	0.609^*∗∗*^	0.346^*∗*^	0.223
Resistin											1	0.407^*∗∗*^	0.296
Adipsin												1	0.284
Total PAI-1													1

^*∗*^ *P* < 0.05.

^*∗∗*^ *P* < 0.01.

**Table 5:** Effect of Eui-E-In-Tang on serum cytokine levels.

Serum cytokine levels (pg/mL)	Study group	*N*	Baseline	Endpoint	*P*
C-peptide	Obese-Eui-E-In-Tang	9	3290.7 ± 1194.0	3481.1 ± 1466.4	0.832
	Obese-placebo	11	3156.3 ± 1444.6	3129.7 ± 2203.9	0.664

GIP	Obese-Eui-E-In-Tang	9	76.3 ± 90.5	124.0 ± 265.9	0.703
	Obese-placebo	11	63.0 ± 48.9	58.6 ± 51.9	0.433

Insulin	Obese-Eui-E-In-Tang	9	57.9 ± 24.2	47.8 ± 25.6	0.262
	Obese-placebo	11	46.3 ± 17.7	91.9 ± 137.8	0.309

Leptin	Obese-Eui-E-In-Tang	9	3749.5 ± 1556.7	3293.5 ± 1701.6	0.037
	Obese-placebo	11	2281.4 ± 1148.6	2856.6 ± 1157.4	0.489

MCP-1	Obese-Eui-E-In-Tang	9	1231.0 ± 381.9	1381.4 ± 473.0	0.471
	Obese-placebo	11	1409.2 ± 615.2	1462.4 ± 760.0	0.767

PP	Obese-Eui-E-In-Tang	9	131.0 ± 100.4	109.5 ± 39.8	0.365
	Obese-placebo	11	193.7 ± 175.0	161.4 ± 196.7	0.401

IL-8	Obese-Eui-E-In-Tang	9	954.3 ± 1610.5	2196.5 ± 4474.1	0.231
	Obese-placebo	11	2473.8 ± 3287.8	3437.3 ± 3850.0	0.494

HGF	Obese-Eui-E-In-Tang	9	299.7 ± 162.5	273.5 ± 166.4	0.373
	Obese-placebo	11	240.3 ± 106.9	268.5 ± 127.4	0.938

Adiponectin	Obese-Eui-E-In-Tang	9	8954.3 ± 1676.9	8551.0 ± 1864.9	0.697
	Obese-placebo	11	9219.6 ± 1097.8	8470.6 ± 1669.0	0.916

Lipocalin-2	Obese-Eui-E-In-Tang	9	4239.4 ± 1258.6	4808.5 ± 2615.8	0.323
	Obese-placebo	11	3465.1 ± 1897.7	4050.4 ± 1833.9	0.440

Resistin	Obese-Eui-E-In-Tang	9	1163.2 ± 763.1	953.9 ± 603.3	0.473
	Obese-placebo	11	940.7 ± 493.1	821.5 ± 415.1	0.556

Adipsin	Obese-Eui-E-In-Tang	9	17645.9 ± 591.6	17110.3 ± 808.6	0.751
	Obese-placebo	11	17553.3 ± 624.6	17067.4 ± 894.1	0.907

Total PAI-1	Obese-Eui-E-In-Tang	9	2867.1 ± 866.5	2566.7 ± 636.9	0.438
	Obese-placebo	11	2556.1 ± 789.0	2858.0 ± 886.5	0.387
